# Supplementary material for: Identification of photoperiod-induced specific miRNAs in the adrenal glands of Sunite sheep (Ovis aries)
Source: Front Vet Sci. 2022 Jul 22;9:888207. doi: 10.3389/fvets.2022.888207 (PMC9354845; doi:10.3389/fvets.2022.888207)
Supplement: Supplementary file 1 [file Data_Sheet_1.ZIP › Supplementary file/miRDeep2 file.zip!/miRDeep2 file/Know_Structure/LP42A1/oar-mir-150.pdf]

miRBase precursor : oar-mir-150
Total read count : 1028
oar-miR-150 read count : 1027
remaining reads : 1

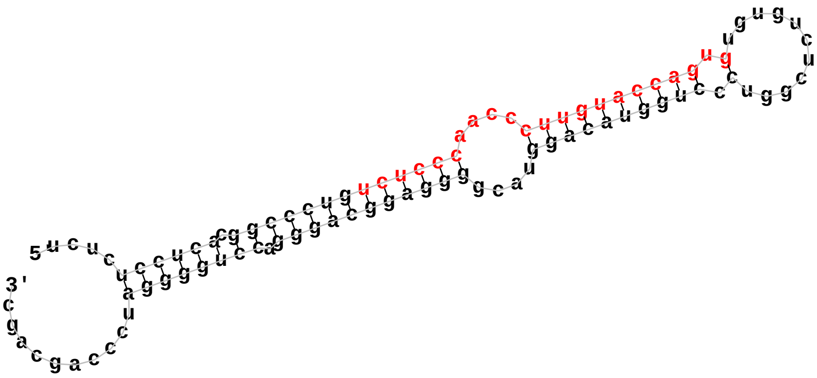

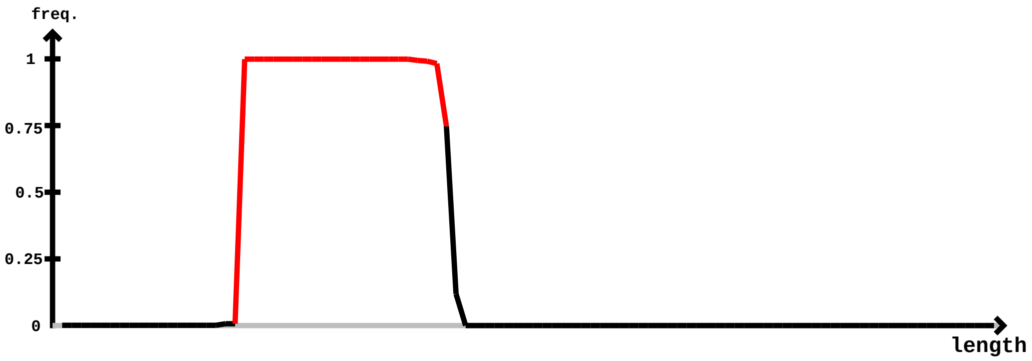


```
                    oar-miR-150
5'- ucucuccucacggcccugucucccaacccuuguaccagugugugucucggucccugguacagguacggggaggcagggaccuggggaucccagcagc   -3'    exp
    ....((((((.(((((((((((((....((((((((((.(...........).))))))))))....))))))))))).))))))))..........    reads    mm    sample
    ucucuccucacggcccug...............................................................................    1        0     seq
    ................ugucucccaacccuuguacca............................................................    1        0     seq
    ................ugucucccaacccuuguaccag...........................................................    1        0     seq
    ................ugucucccaacccuuguaccagu..........................................................    4        0     seq
    ..................ucucccaacccuuguacc.............................................................    5        0     seq
    ..................ucucccaacccuuguacca............................................................    2        0     seq
    ..................ucucccaacccuuguaccag...........................................................    8        0     seq
    ..................ucucccaacccuuguaccagu..........................................................    238      0     seq
    ..................ucucccaacccuuguaccagug.........................................................    646      0     seq
    ..................ucucccaacccuuguaccagugu........................................................    122      0     seq
```
